# Supplementary figures and images for: A hybrid stochastic model of the budding yeast cell cycle
Source: NPJ Syst Biol Appl. 2020 Mar 27;6:7. doi: 10.1038/s41540-020-0126-z (PMC7101447; doi:10.1038/s41540-020-0126-z)

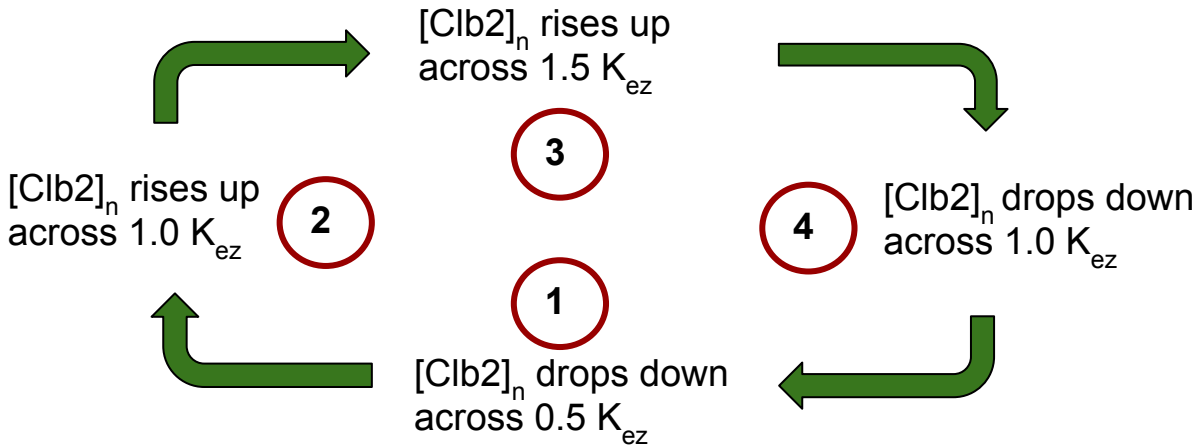

Supplement: Supplementary file 3 — Supp Code [file 41540_2020_126_MOESM3_ESM.zip › Supplementary File/Supplementary_Source_tex/events.pdf]
